# Does socioeconomic status modify how individuals perceive or describe their own health? An assessment of reporting heterogeneity in the Health Survey for England

**DOI:** 10.1136/bmjph-2023-000813

**Published:** 2024-09-10

**Authors:** Rosanna Fforde, Nicholas Parsons, Oyinlola Oyebode

**Affiliations:** 1 Warwick Medical School, University of Warwick, Coventry, UK; 2 Clinical Trials Unit, Warwick Medical School, University of Warwick, Coventry, UK; 3 Division of Health Sciences, Warwick Medical School, University of Warwick, Coventry, UK; 4 Faculty of Medicine and Dentistry, Wolfson Institute of Population Health, Queen Mary University of London, London, UK

**Keywords:** public health, sociodemographic factors, social medicine

## Abstract

**Background:**

Self-reported health (SRH) is widely used as a proxy for health status; it is a simple, holistic measure of health and has been associated with other health outcomes. However, variation in these associations has been found by subgroup, leaving open the possibility of systematic reporting differences, including by socioeconomic status.

**Methods:**

Using data from the 2017 and 2018 waves of the Health Survey for England, we assessed the relationship between deprivation quintile and SRH using multiple linear regression models for EQ-5D score, a health-related quality of life measure. Reporting heterogeneity between deprivation quintiles was assessed by the significance of model interaction terms. Analyses were stratified by sex and accounted for age group, ethnicity, marital status and religion (females only).

**Results:**

Significant interactions were found between deprivation quintile and SRH in the models for EQ-5D score for participants reporting poor health in the two most deprived quintiles, with coefficients ranging from –0.041 to –0.098. The largest differences were in the second most deprived quintile for men (–0.075, 95% CI: –0.110 to –0.040, p<0.001) and the most deprived quintile for women (–0.098, 95% CI: –0.128 to –0.067, p<0.001). Secondary analyses using body mass index as the response variable, for models structured in a similar way to those for EQ-5D, found no significant interaction terms between deprivation quintile and SRH.

**Conclusions:**

This analysis suggests that people from more deprived areas who report themselves to be in poor health may have worse health, as measured by EQ-5D, than those from less deprived areas. This could lead to an underestimation of health inequalities, including in measures, such as healthy life expectancy in England, that incorporate SRH data.

**WHAT IS ALREADY KNOWN ON THIS SUBJECT**

**WHAT THIS STUDY ADDS**

- This is the first UK study to use a health utility measure as a benchmark for ‘objective’ health in assessing reporting differences by socioeconomic status, and the first to use the Index of Multiple Deprivation, facilitating its practical application, as the latter is widely used for measuring health inequalities in England.
- It finds that people who live in the most deprived two quintiles of England who report poor health have lower health utility scores than would be expected based on their deprivation level and SRH status.

**HOW THIS STUDY MIGHT AFFECT RESEARCH, PRACTICE OR POLICY**

- This study implies that using SRH as a proxy for health status could potentially underestimate health inequalities by socioeconomic status, and hence further research may wish to quantify what impact this may have on healthy life expectancy estimates and, potentially, public health resourcing.

## Introduction

 A single-question measure of self-reported health (SRH) has been widely used in health research globally. Variations exist, but the question typically asks: ‘How is your health in general? Would you say it is: very good, good, fair, bad, or very bad?’ Despite its simplicity, a range of associations with health outcomes have been found, including with mortality.^1 2^

SRH is also used to inform policy; for example, in England, it is used as the basis for the health component of healthy life expectancy, a measure summarising the health of populations relative to their life expectancy.^3^ Nonetheless, variation has been found in the magnitude (or existence) of associations by subgroup, suggesting that SRH may not mean the same for all groups.^14^,^6^

As Adams and White highlight, SRH may incorporate expectations about what ‘good’ or ‘bad’ health is and an assessment of how one’s own health relates to this.^7^ Given the known relationship between health and socioeconomic status—with gaps in life expectancy of 9.7 years for men and 8.0 years for women between the 10% most and least deprived areas in England in 2018–20^8^—and qualitative research that also highlights how marginalised groups have lower health expectations,^9^ it is plausible that those from deprived areas may have a higher threshold for reporting poor health. This could lead to underestimates of health inequalities when SRH is used as a proxy for health.

However, disentangling differences in health perception and underlying health state are not straightforward. One response to this is to use SRH solely as a measure of people’s perception and reporting of health.^10^ However, this does not stop it being widely used in practice as a proxy for health status,^11^ with implications for resource allocation.^5 12^ In order to assess reporting heterogeneity, studies have generally looked for variation or interactions between SRH and one or more alternative health measures by socioeconomic status. Alternative health measures have included biomarkers,^7 11 13^ health utility values^5 14^ and self-reported health conditions/functional limitations, either separately^6^ or as part of a synthetic index.^12^

Findings have been mixed, with several studies suggesting the health of those with lower socioeconomic status is worse for the same level of SRH, but within papers this has depended on the health measure, sex, socioeconomic group and the level of health reported.^67 11^,^13^ Layes *et al* reported that those in the top two income deciles had better health utility values than their SRH level would predict, while the three lowest income deciles had lower health utility values than their SRH level would predict.^5^ Lindeboom and van Doorslaer did not find, overall, that income or education modified SRH.^14^

To our knowledge, only one study has previously used the Health Survey for England to explore reporting heterogeneity in SRH^7^ and none has used a health utility measure to explore this within a UK setting. This study aims to update and extend previous research by looking at the relationship between SRH and a health utility measure, EQ-5D, by socioeconomic status and sex in England.

## Methods

We used a health-related quality of life measure, EQ-5D, as a proxy for ‘objective’ health to explore whether differences in SRH by deprivation reflect only ‘true’ differences in health status or whether reporting heterogeneity might modify this. We tested the hypothesis that if there were no reporting heterogeneity, no interaction between deprivation quintile and SRH would be seen in a linear regression model for EQ-5D utility score. Secondary analyses used body mass index (BMI) as the response variable. We also performed descriptive analyses to characterise the relationship between SRH, deprivation and other sociodemographic variables.

### Data

This study uses data from the 2017 and 2018 waves of the Health Survey for England, a nationally representative, annual cross-sectional survey.^15 16^ It is commissioned nationally and each wave consists of a core set of questions/measures in addition to some which are wave-specific. The design and methods used in the 2017 and 2018 waves are summarised below, but full details are available in the relevant Methods reports.^17 18^

Each wave used a random stratified sampling design, first selecting primary sampling units based on postcode sectors, and then selecting addresses within these.^17 18^ Only one household per address was selected; this was chosen randomly for addresses with multiple dwelling units and/or households. Within each household, all adults (up to 10) were eligible for interview and up to four children, based on their ages. Interviews were face-to-face and used computer-assisted personal interviewing, consisting of a household questionnaire and an individual questionnaire, and a self-completion booklet. Participants were also weighed and measured at this interview. A subsequent nurse visit was also offered to all participants in 2017 and a subset of households in 2018, but no variables from this visit were included in our analysis. Fieldwork took place across the year in question and up to March of the following year (ie, January 2017 to March 2018 for the 2017 wave). Only private households are covered by the Health Survey for England and not institutional settings.

The 2017 and 2018 waves were chosen as they reflect two consecutive years when EQ-5D data were collected, as these are not collected in every wave, and the same version of the Index of Multiple Deprivation (IMD) was available. The use of two successive waves created a larger sample for analysis.

### Variables

EQ-5D-5L (EQ-5D) is a health-related quality of life measure developed by EuroQol^19^ consisting of five domains (mobility, self-care, usual activities, pain/discomfort and anxiety/depression) that are assessed using five levels of response (none, slight, moderate, severe and extreme, scored as 1–5). EQ-5D is the preferred health utility tool of the National Institute for Health and Care Excellence (NICE), a body that provides recommendations on which medicines/treatments should be publicly funded in England and Wales.

We mapped the profile of responses (eg, 11111) using van Hout *et al*’s ‘crosswalk’ approach to a three-level value set derived from a UK population, as recommended by NICE at the time analysis on this study began.^20^,^22^ The values provide a population-based judgement of where each profile sits on a scale of –0.594 to 1, where 1 equates to optimal health and 0 notionally to death. Negative values are possible where a state is felt to be worse than death.

As EQ-5D is self-reported, one potential criticism is that it may itself be subject to reporting heterogeneity.^11^ However, Layes *et al* also used a health utility measure (the Health Utility Index Mark 3) to assess reporting heterogeneity in SRH on the basis that it covers a holistic view of health, the questions relate to functional measures of health, which may reduce subjectivity, and that the value set that responses are mapped to has been derived from the population rather than the individual in question.^5^ Nonetheless, to explore this potential limitation, we conducted secondary analyses using BMI (weight in kilograms divided by the square of height in metres) as the response variable instead of EQ-5D, on the basis that BMI can be objectively measured, and provides an informative complementary analysis to that provided by EQ-5D.

SRH was assessed via the question: ‘How is your health in general? Would you say it was…very good, good, fair, bad or very bad?’ We dichotomised responses into very good and good (referred to as ‘good’), and fair, bad, and very bad (referred to as ‘poor’). While dichotomisation results in a loss of information, this is how SRH is used in practice for the healthy life expectancy metric in England^3^ and hence this cut-point is relevant for understanding policy implications.

Socioeconomic status was measured using 2015 IMD quintiles.^23^ IMD is a composite, place-based measure of deprivation that is widely used for the measurement of health inequalities in England. The most deprived quintile indicates that the participant lived in the upper 20% most deprived neighbourhoods in England.

Age group, ethnicity, marital status, and religion were included as covariates in models.

Table 1 provides further details on how data were collected for each variable.

**Table 1:** Variables in analysis^17 18 28 29^

Variable type	Variable	Data source within Health Survey for England
Response	EQ-5D-5L	Self-completion booklet. Note: responses were mapped to a UK value set
	BMI (secondary analysis)	Height and weight measurements were taken by a trained interviewer, using a portable stadiometer (to nearest even millimetre) and Seca 877 digital weighing scales (to nearest 100 g)
Explanatory	SRH	Individual questionnaire
	IMD	Derived from address
	Age group	Age collected from household reference person in household questionnaire and verified in individual questionnaire
	Ethnicity	Individual questionnaire
	Marital status	Collected from household reference person in household questionnaire
	Religion	Self-completion booklet

BMIbody mass indexIMDIndex of Multiple DeprivationSRHself-reported health

### Analysis

To test whether deprivation quintile interacted with SRH, multiple linear regression models for EQ-5D score were fitted, assuming approximate normality for EQ-5D scores which is consistent with modelling approaches used elsewhere, with (preplanned) separate models for males and females. Terms were added using a forward selection methodology; variables were added to a base model initially including only age group and models of increasing complexity were compared using likelihood ratio tests (LRTs) to the previous (less complex) model to assess whether adding the term(s) had improved the overall model fit. The final models included age, ethnicity, marital status, religion (females only), IMD quintile, SRH, and the interaction between IMD quintile and SRH. The same approach to model fitting was used for the secondary analysis with BMI as the response variable.

The regression analyses were stratified by sex; there is reason to believe that reporting heterogeneity in SRH may differ by sex^14^ and this has been the approach taken in other studies.^6 11^

The descriptive analyses were directly age standardised using an average of the 2017 and 2018 Office for National Statistics mid-year population estimates for England and a final age group of 85 years and above. These descriptive analyses were weighted using the weights recommended by the Health Survey for England, to allow generalisability to the English population. Weights were not used in the linear regression models as the analysis did not attempt to create nationally representative prevalence estimates.

All analyses were implemented in R (V.4.1.2 and V.4.2.3)^24^ and assessed significance at the 5% level.

### Missing data

As EQ-5D was only collected from those aged 16 years and above, we excluded children under the age of 16 from our analysis, leaving a potential sample of 16 175 participants. Participants with missing data on variables of interest were excluded, with the analysis using an assumption that complete cases were, all other things being equal, the same as cases with missing data: that is, data were missing at random. These exclusions were made prior to models being fitted. All participants had data on age group, sex and IMD quintile, while data for SRH, marital status and ethnicity were highly complete (each greater than 99.5%, with SRH and marital status missing data from fewer than five participants each). EQ-5D and religion, which were both collected through a self-completion booklet, were less complete: 11% of participants were missing EQ-5D and 11% were missing religion. After exclusions were made for those with incomplete SRH, EQ-5D, marital status, ethnicity or religion, the final sample for analysis was 14 116 (87%). Due to the overlap between those missing EQ-5D and religion, the added impact of excluding religion after all other exclusions was only 1.7% of the sample aged 16 and above. Participant characteristics are summarised in online supplemental appendix table A.1. Due to missing BMI data, the sample used for the secondary analysis was 12 182 (75%).

### Patient and public involvement

No patients or members of the public were involved in the design or completion of this study.

## Results

### Descriptive analysis

Weighted, age-standardised percentages of participants reporting good health by deprivation quintile and various characteristics are shown in table 2. Some categories were grouped for this analysis and BMI was categorised to allow for age-standardisation.

**Table 2:** Weighted and age-standardised proportion of participants reporting good health

% in good health	Total	Deprivation quintile					Difference between quintiles 1 and 5 (% pts)
		1 (least deprived)	2	3	4	5 (most deprived)	
All participants	75.7	82.9	80.1	76.8	71.5	63.9	19.0
**Sex** *							
Male	77.0	84.0	81.0	78.4	73.0	66.2	17.8
Female	74.5	81.9	79.1	75.5	70.2	61.8	20.1
**Age**							
16–19	86.6	90.6	88.5	88.4	87.3	80.0	10.6
20–24							
25–29							
30–34							
35–39	79.4	86.3	84.3	77.6	77.4	71.2	15.1
40–44							
45–49							
50–54	71.7	81.9	77.0	75.6	63.6	55.3	26.6
55–59							
60–64							
65–69	61.3	70.2	67.3	61.8	52.0	43.2	27.0
70–74							
75–79							
80–84							
85–89							
90+							
**Ethnicity**							
White	75.9	83.1	79.8	76.8	71.3	63.2	19.9
Black	72.2	76.0	80.5	76.6	71.6	62.6	13.4
Asian							
Mixed/multiple ethnic background							
Any other ethnic group							
**Marital status**							
Single	68.2	79.5	75.0	68.7	64.6	59.3	20.2
Separated							
Divorced							
Widowed							
Married	77.8	84.6	81.7	79.7	73.7	62.8	21.8
Cohabitees							
**Religion**							
No religion	76.4	82.9	79.7	78.1	73.8	62.8	20.1
Christian—Catholic	75.9	83.0	79.8	77.6	70.7	65.6	17.4
Christian—all other denominations							
Buddhist							
Hindu							
Jewish							
Muslim							
Sikh							
Any other religion							
**BMI** †							
Underweight‡	–	–	–	–	–	–	–
Normal weight	83.1	88.9	86.6	84.4	77.9	70.4	18.5
Overweight	80.3	83.9	85.3	81.4	75.8	69.4	14.5
Obese	66.7	77.1	70.2	69.1	64.3	57.2	19.9
Missing	65.5	72.3	71.6	64.2	63.6	54.7	17.6

* Figures are not age-standardised separately by sex (ie, against separate male/female standard populations), except for ‘Sex’ where sex-specific standard populations are used.

† BMI was classified as: underweight <18.5; normal weight 18.5–<25; obese 25<–30; obese ≥30.

‡ It was not possible to age-standardise the proportion of underweight people in good health due to small numbers.

BMIbody mass index

As expected, there was a downward trend in self-reported good health with increasing deprivation, from 82.9% in the least deprived quintile to 63.9% in the most deprived quintile. This increased in each successive age group such that in those aged 65 years and above, the proportion reporting good health in quintile 1 was 1.6 times that of quintile 5 (70.2% vs 43.2%). In participants aged 50–64 years, below the age at which you can draw a state pension in the UK, 81.9% of those in quintile 1 reported good health compared with 55.3% in quintile 5. The former is similar to or slightly higher than the 80.0% of 16–34-year olds reporting good health in quintile 5.

A similar but slightly smaller proportion of females reported good SRH relative to males (74.5% vs 77.0%), while a smaller proportion of those who were single, separated, divorced or widowed reported good health relative to those who were married or cohabiting (68.2% vs 77.8%). Although the BMI group with the highest proportion reporting good health was the normal weight group (83.1%), this was similar to the overweight group (80.3%). In contrast, the proportion reporting good health in the obese group was lower (66.7%).

### Linear regression

Linear regression models for EQ-5D utility scores were fitted, with separate models for males and females, including age, ethnicity, marital status, IMD quintile, SRH and the interaction between IMD quintile and SRH (table 3). Religion was included in the final female but not male model as including this in the latter did not improve fit.

**Table 3:** Linear regression models predicting EQ-5D score

Variable	Males				Females			
	Estimate	95% CI		P value	Estimate	95% CI		P value
		Lower	Upper			Lower	Upper	
Intercept	0.946	0.921	0.971	<0.001	0.910	0.885	0.934	<0.001
**Age**								
16–19	–				–			
20–24	–0.026	–0.057	0.005	0.101	0.010	–0.019	0.040	0.496
25–29	–0.037	–0.068	–0.006	0.020	–0.007	–0.036	0.022	0.630
30–34	–0.024	–0.054	0.007	0.129	0.001	–0.027	0.030	0.917
35–39	–0.058	–0.088	–0.027	<0.001	–0.015	–0.043	0.013	0.307
40–44	–0.055	–0.085	–0.024	<0.001	–0.019	–0.048	0.009	0.182
45–49	–0.080	–0.110	–0.050	<0.001	–0.039	–0.068	–0.011	0.007
50–54	–0.065	–0.096	–0.035	<0.001	–0.041	–0.070	–0.012	0.005
55–59	–0.076	–0.106	–0.046	<0.001	–0.036	–0.065	–0.007	0.016
60–64	–0.094	–0.125	–0.064	<0.001	–0.056	–0.086	–0.027	<0.001
65–69	–0.085	–0.115	–0.054	<0.001	–0.056	–0.086	–0.026	<0.001
70–74	–0.071	–0.102	–0.039	<0.001	–0.050	–0.080	–0.020	0.001
75–79	–0.094	–0.128	–0.060	<0.001	–0.061	–0.094	–0.029	<0.001
80–84	–0.101	–0.137	–0.064	<0.001	–0.062	–0.098	–0.026	<0.001
85–89	–0.131	–0.175	–0.088	<0.001	–0.118	–0.160	–0.076	<0.001
90+	–0.186	–0.247	–0.125	<0.001	–0.144	–0.198	–0.089	<0.001
**Ethnicity**								
White	–				–			
Black	0.030	0.000	0.060	0.050	0.032	0.008	0.056	0.009
Asian	0.017	–0.002	0.035	0.075	0.021	–0.005	0.047	0.107
Mixed/multiple ethnic background	–0.004	–0.045	0.038	0.865	0.009	–0.025	0.043	0.596
Any other ethnic group	0.004	–0.047	0.055	0.878	–0.006	–0.055	0.044	0.826
**Marital status**								
Single	–				–			
Married	0.036	0.022	0.051	<0.001	0.030	0.016	0.044	<0.001
Separated	0.013	–0.027	0.052	0.533	0.002	–0.028	0.032	0.884
Divorced	0.003	–0.021	0.027	0.814	–0.024	–0.044	–0.005	0.013
Widowed	0.011	–0.018	0.040	0.462	0.010	–0.012	0.031	0.365
Cohabitees	0.038	0.021	0.056	<0.001	0.031	0.015	0.047	<0.001
**Religion**								
No religion					–			
Christian—Catholic					0.014	0.002	0.026	0.023
Christian—all other denominations					0.000	–0.010	0.011	0.939
Buddhist					0.016	–0.044	0.076	0.607
Hindu					–0.010	–0.052	0.032	0.637
Jewish					0.074	0.009	0.138	0.025
Muslim					–0.037	–0.065	–0.009	0.011
Sikh					–0.013	–0.071	0.045	0.661
Any other religion					–0.020	–0.056	0.016	0.276
**IMD quintile**								
1 (least deprived)	–				–			
2	–0.006	–0.022	0.010	0.463	–0.004	–0.019	0.010	0.545
3	–0.009	–0.025	0.008	0.296	–0.015	–0.030	0.000	0.046
4	–0.019	–0.036	–0.002	0.026	–0.024	–0.039	–0.009	0.002
5 (most deprived)	–0.018	–0.036	0.000	0.047	–0.019	–0.035	–0.003	0.019
**Self-reported health**								
Good	–				–			
Poor	–0.214	–0.240	–0.188	<0.001	–0.231	–0.255	–0.208	<0.001
**Interaction: IMD quintile and self-reported health**								
1/good	–				–			
2/poor	0.008	–0.027	0.043	0.653	–0.029	–0.060	0.003	0.079
3/poor	–0.009	–0.044	0.026	0.615	–0.027	–0.059	0.005	0.098
4/poor	–0.075	–0.110	–0.040	<0.001	–0.046	–0.076	–0.015	0.004
5/poor	–0.041	–0.076	–0.006	0.020	–0.098	–0.128	–0.067	<0.001

Adjusted R-squared for males: 0.292.

Adjusted R-squared for females: 0.346.

IMDIndex of Multiple Deprivation

Adding an interaction term between IMD quintile and SRH increased the explanatory power of the model for both males and females relative to models without this (LRT, p<0.001). For both males and females, the two most deprived quintiles showed significant interactions with SRH. The coefficients indicate that the individuals in those two quintiles who reported themselves to be in poor health have a lower EQ-5D score than would be predicted from their deprivation quintile and SRH status alone.

The magnitude of these differences in EQ-5D score ranged from –0.041 to –0.098. The largest difference for men was in the second most deprived quintile (–0.075, 95% CI: –0.110 to –0.040, p<0.001), which is similar to the impact of age for 55–59-year olds relative to 16–19-year olds (–0.076). The largest difference for women was in the most deprived quintile (–0.098, 95% CI: –0.128 to –0.067, p<0.001), which is higher in magnitude than the impact of age in all age groups up to and including those aged 80–84 years relative to those aged 16–19 years (–0.062).

As a secondary analysis, we fitted models with BMI as the response variable (table 4). Unlike the EQ-5D models, the male model with the deprivation and SRH interaction term was not a better fit than one without it (LRT, p=0.335), although the female one was (LRT, p=0.006). Although poor SRH and deprivation were predictors of higher BMI, none of the categories in the interaction term showed significant results for males or females.

**Table 4:** Linear regression models predicting BMI

Variable	Males				Females			
	Estimate	95% CI		P value	Estimate	95% CI		P value
		Lower	Upper			Lower	Upper	
Intercept	22.911	22.229	23.593	<0.001	22.222	21.370	23.075	<0.001
**Age**								
16–19	–				–			
20–24	1.314	0.462	2.165	0.003	1.955	0.933	2.978	<0.001
25–29	1.955	1.110	2.799	<0.001	2.626	1.632	3.619	<0.001
30–34	2.181	1.349	3.013	<0.001	3.125	2.150	4.101	<0.001
35–39	2.422	1.582	3.262	<0.001	3.154	2.184	4.124	<0.001
40–44	3.071	2.239	3.904	<0.001	2.868	1.881	3.854	<0.001
45–49	3.889	3.059	4.719	<0.001	3.908	2.926	4.890	<0.001
50–54	3.855	3.022	4.688	<0.001	4.209	3.215	5.203	<0.001
55–59	3.980	3.153	4.806	<0.001	3.837	2.833	4.842	<0.001
60–64	3.769	2.923	4.615	<0.001	2.995	1.961	4.029	<0.001
65–69	3.339	2.490	4.189	<0.001	3.729	2.689	4.768	<0.001
70–74	3.018	2.153	3.883	<0.001	3.157	2.107	4.207	<0.001
75–79	2.708	1.779	3.637	<0.001	2.769	1.634	3.904	<0.001
80–84	2.371	1.345	3.397	<0.001	1.583	0.284	2.882	0.017
85–89	0.996	–0.256	2.248	0.119	1.381	–0.198	2.960	0.087
90+	1.895	–0.109	3.899	0.064	0.143	–2.006	2.292	0.896
**Ethnicity**								
White	–				–			
Black	–1.205	–2.021	–0.389	0.004	1.865	1.030	2.699	<0.001
Asian	–1.463	–1.977	–0.949	<0.001	–1.317	–2.201	–0.432	0.004
Mixed/multiple ethnic background	–0.364	–1.505	0.777	0.532	–0.928	–2.088	0.233	0.117
Any other ethnic group	–1.691	–3.077	–0.305	0.017	–0.480	–2.104	1.145	0.563
**Marital status**								
Single	–				–			
Married	1.721	1.305	2.136	<0.001	0.364	–0.121	0.849	0.141
Separated	2.003	0.912	3.094	<0.001	1.089	0.083	2.094	0.034
Divorced	1.185	0.498	1.871	<0.001	–0.029	–0.704	0.646	0.933
Widowed	2.265	1.432	3.099	<0.001	1.336	0.567	2.104	<0.001
Cohabitees	1.101	0.616	1.587	<0.001	0.338	–0.224	0.899	0.239
**Religion**								
No religion					–			
Christian—Catholic					0.234	–0.177	0.645	0.265
Christian—all other denominations					1.092	0.718	1.465	<0.001
Buddhist					–1.477	–3.699	0.746	0.193
Hindu					0.655	–0.806	2.116	0.380
Jewish					–1.414	–3.779	0.952	0.241
Muslim					1.223	0.267	2.179	0.012
Sikh					0.725	–1.207	2.658	0.462
Any other religion					1.217	–0.070	2.503	0.064
**IMD quintile**								
1 (least deprived)	–				–			
2	0.255	–0.176	0.686	0.247	0.314	–0.178	0.805	0.211
3	0.334	–0.109	0.776	0.140	1.145	0.637	1.654	<0.001
4	0.760	0.295	1.224	0.001	1.249	0.728	1.769	<0.001
5 (most deprived)	1.334	0.845	1.823	<0.001	2.279	1.726	2.831	<0.001
**Self-reported health**								
Good	–				–			
Poor	1.825	1.073	2.578	<0.001	2.785	1.931	3.639	<0.001
**Interaction: IMD quintile and self-reported health**								
1/good	–				–			
2/poor	0.400	–0.599	1.399	0.433	0.941	–0.207	2.088	0.108
3/poor	–0.499	–1.503	0.506	0.331	0.465	–0.680	1.611	0.426
4/poor	–0.432	–1.429	0.565	0.396	0.338	–0.764	1.441	0.548
5/poor	–0.201	–1.192	0.790	0.690	–0.938	–2.044	0.168	0.096

Adjusted R-squared for males: 0.116.

Adjusted R-squared for females: 0.095.

BMIbody mass indexIMDIndex of Multiple Deprivation

Further EQ-5D models that included an interaction term between age and SRH did show an improvement relative to models without it for both males and females (online supplemental appendix table A.2). However, the interaction between the two most deprived quintiles and SRH remained significant for both males and females and this did not have a large effect on the magnitude of the coefficients (range: –0.004 to 0.003).

## Discussion

This is the first study to assess reporting heterogeneity in SRH in the Health Survey for England using IMD as a measure of socioeconomic status and EQ-5D as a reference standard for health status. It also provides further descriptive analysis on the association between SRH and deprivation and how this varies by population characteristics.

We found that there was a statistically significant interaction between SRH and socioeconomic status for the relationships between those in the most deprived two quintiles who reported poor health, relative to those in the least deprived quintile reporting good health. The coefficients indicated that the EQ-5D score for these two quintiles (for both males and females) was lower when this additional information was included in the model (–0.041 to –0.098). The minimum clinically important difference for EQ-5D for the three-level value set used by this study has been estimated at 0.074,^25^ and two of the four findings meet this threshold: males in the second most deprived quintile (–0.075) and females in the most deprived quintile (–0.098). For the former, the additional effect on EQ-5D is similar to being aged 55–59 years (relative to being aged 16–19 years), whereas for the latter, this effect was greater than the impact of age for age groups up to and including 80–84 (relative to the 16–19 age group).

Furthermore, a summary of the ‘disutility’ or change in EQ-5D value associated with various conditions suggests that the interaction effects we found are in the same range as the disutility associated with asthma (–0.046), acute myocardial infarction (–0.063), diabetes (–0.071) or neurotic disorders (–0.094).^26^ A further study using Health Survey for England data estimated the impact of smoking 20 or more cigarettes a day (relative to never smoking) on EQ-5D as –0.062 and the impact of being morbidly obese (BMI≥40, relative to a BMI of 18.5 to <25) as –0.105.^27^ Given their similarity in size to those associated with known health issues and risk factors, this suggests that the magnitude of our findings may be meaningful.

The models predicting BMI did not show a significant interaction between SRH and deprivation. One explanation may be that although BMI is an objective measure, it is not necessarily a proxy for overall health; people may be unwell and have a normal BMI, and those with a raised BMI may be at greater risk of ill health but not yet be experiencing this. These models were also not a good fit, with adjusted R-squared values of 0.12 for males and 0.10 for females. SRH and deprivation (for the two/three most deprived deciles, depending on sex) were nonetheless independently associated with increased BMI.

This study’s strengths were that it used a large sample from a nationally representative survey and used the IMD as its measure of socioeconomic status. Although this is a place-based measure rather than a person-based one, it is widely used in the study of health inequalities in England, facilitating the interpretation of the results into practice. It was also available for all participants, reducing the risk of response bias or the need to limit the age range to account for those who might not have finished their studies or for whom occupational status may be less accurate, which has been a feature of other studies on reporting heterogeneity in SRH.^5^,^712 13^ Full data on all variables of interest were available for 87% of survey participants aged 16 and above, supporting its generalisability to English settings.

One potential limitation was the use of EQ-5D; as this is self-reported, it could be vulnerable to the same bias under investigation. This would mean our analyses underestimate the true difference in objective health for a given subjective health. However, as discussed above, there is reason to believe that if there is such a bias, it might not be so acute due to the focus of the questions on functional limitations and the use of a population-based value set.^5^ Nonetheless, future research may wish to explore alternative, more objectively defined, measures of health. A second limitation is that the models explained only around 30%–35% of the variation in EQ-5D. This could suggest that there is not a good match between the health concepts described by EQ-5D and SRH, or that SRH, as a categorical variable, and particularly in its dichotomised form, does not contain enough information to be able to explain the variation in EQ-5D values. One explanation for our findings could be that the distribution of responses within ‘poor’ health (fair/bad/very bad) may be more concentrated towards the very bad health end of the spectrum in participants from more deprived areas and hence that use of the full range of responses may not show reporting heterogeneity. Nonetheless, as the dichotomised version is what is used in practice for healthy life expectancy in England, our findings remain relevant. Further analysis may wish to explore reporting heterogeneity by SRH state.

This study implies that the use of healthy life expectancy measures based on SRH could potentially underestimate inequalities based on socioeconomic deprivation. Further study to quantify any impact on healthy life expectancy at area level would be of value to understand the policy implications. However, even if there is an element of underestimation, SRH remains a powerful tool for evidencing health inequalities in England, such as in our descriptive analyses—where 50–64-year olds in the least deprived quintile reported similar or slightly better health than 16–34-year olds in the most deprived quintile—and the simplicity of the SRH question has enabled it to be incorporated into routine, large-scale surveys, allowing monitoring of geographical inequalities and change over time.

## supplementary material

10.1136/bmjph-2023-000813 online supplemental file 1

## Data Availability

Data may be obtained from a third party and are not publicly available.
