# Supplementary material for: Does socioeconomic status modify how individuals perceive or describe their own health? An assessment of reporting heterogeneity in the Health Survey for England
Source: BMJ Public Health. 2024 Sep 10;2(2):e000813. doi: 10.1136/bmjph-2023-000813 (PMC11816828; doi:10.1136/bmjph-2023-000813)
Supplement: online supplemental file 1 [file bmjph-2-2-s001.pdf]

Appendix: Additional tables

Table A.1: Participant characteristics

Variable	N	%
**Sex**		
Male	6,189	43.8
Female	7,927	56.2
**Age**		
16–34	3,150	22.3
35–49	3,528	25.0
50–64	3,625	25.7
65+	3,813	27.0
**Ethnicity**		
White	12,423	88.0
All other ethnic groups	1,693	12.0
**Religion**		
Any religion	8,993	63.7
No religion	5,123	36.3
**Marital status**		
Married/cohabiting	9,265	65.6
Single/separated/divorced/widowed	4,851	34.4
**IMD quintile**		
1 (least deprived)	2,840	20.1
2	3,028	21.5
3	2,826	20.0
4	2,850	20.2
5 (most deprived)	2,572	18.2
**Self-reported health**		
Good	10,464	74.1
Poor	3,652	25.9
**EQ-5D**		
Mean	0.823	–

Table A.2: Linear regression models predicting EQ-5D score with additional age group–SRH interaction term

Variable	Males				Females			
	Estimate	95% confidence interval		*P*-value	Estimate	95% confidence interval		*P*-value
		Lower	Upper			Lower	Upper	
Intercept	0.934	0.909	0.960	<0.001	0.901	0.875	0.927	<0.001
**Age**								
16–19	–				–			
20–24	–0.023	–0.056	0.010	0.167	0.001	–0.031	0.033	0.944
25–29	–0.026	–0.058	0.007	0.120	0.003	–0.028	0.034	0.842
30–34	–0.024	–0.056	0.008	0.141	0.006	–0.025	0.036	0.716
35–39	–0.047	–0.080	–0.015	0.004	–0.011	–0.041	0.019	0.457
40–44	–0.046	–0.078	–0.014	0.005	–0.011	–0.042	0.020	0.479
45–49	–0.059	–0.091	–0.027	<0.001	–0.028	–0.059	0.003	0.079
50–54	–0.063	–0.096	–0.031	<0.001	–0.020	–0.051	0.011	0.209
55–59	–0.057	–0.089	–0.025	<0.001	–0.014	–0.046	0.017	0.373
60–64	–0.064	–0.097	–0.031	<0.001	–0.033	–0.066	–0.001	0.044
65–69	–0.069	–0.102	–0.035	<0.001	–0.039	–0.071	–0.006	0.020
70–74	–0.067	–0.102	–0.033	<0.001	–0.054	–0.086	–0.021	0.001
75–79	–0.081	–0.118	–0.044	<0.001	–0.068	–0.105	–0.031	<0.001
80–84	–0.093	–0.135	–0.052	<0.001	–0.075	–0.119	–0.031	<0.001
85–89	–0.117	–0.170	–0.064	<0.001	–0.140	–0.193	–0.088	<0.001
90+	–0.200	–0.278	–0.122	<0.001	–0.150	–0.218	–0.082	<0.001
**Ethnicity**								
White	–				–			
Black	0.030	–0.001	0.060	0.054	0.031	0.007	0.054	0.012
Asian	0.016	–0.002	0.035	0.087	0.021	–0.004	0.047	0.104
Mixed/multiple ethnic background	–0.003	–0.044	0.039	0.902	0.010	–0.023	0.044	0.546
Any other ethnic group	0.006	–0.045	0.056	0.828	–0.008	–0.057	0.041	0.760
**Marital status**								
Single	–				–			
Married	0.036	0.021	0.051	<0.001	0.029	0.016	0.043	<0.001
Separated	0.012	–0.027	0.051	0.553	0.005	–0.025	0.035	0.737
Divorced	0.002	–0.022	0.027	0.854	–0.023	–0.042	–0.003	0.021
Widowed	0.010	–0.018	0.039	0.478	0.010	–0.012	0.031	0.367
Cohabitees	0.039	0.021	0.056	<0.001	0.030	0.014	0.047	<0.001
**Religion**								
No religion					–			
Christian – Catholic					0.013	0.001	0.025	0.030
Christian – all other denominations					0.001	–0.010	0.012	0.835
Buddhist					0.016	–0.044	0.076	0.601
Hindu					–0.010	–0.052	0.033	0.655
Jewish					0.076	0.012	0.140	0.020
Muslim					–0.035	–0.063	–0.007	0.014
Sikh					–0.015	–0.073	0.042	0.600
Any other religion					–0.021	–0.057	0.014	0.241
**IMD quintile**								
1 (least deprived)	–				–			
2	–0.006	–0.021	0.010	0.485	–0.005	–0.019	0.010	0.533
3	–0.008	–0.024	0.008	0.338	–0.015	–0.030	0.000	0.049
4	–0.018	–0.035	–0.001	0.040	–0.023	–0.038	–0.008	0.003
5 (most deprived)	–0.017	–0.034	0.001	0.070	–0.018	–0.034	–0.002	0.029
**Self-reported health**								
Good	–				–			
Poor	–0.087	–0.172	–0.002	0.046	–0.172	–0.242	–0.103	<0.001
**Interaction: IMD quintile and self-reported health**								
1/Good	–				–			
2/Poor	0.006	–0.029	0.041	0.753	–0.025	–0.056	0.007	0.131
3/Poor	–0.005	–0.040	0.030	0.765	–0.024	–0.056	0.008	0.140
4/Poor	–0.075	–0.110	–0.041	<0.001	–0.042	–0.073	–0.011	0.008
5/Poor	–0.045	–0.080	–0.009	0.013	–0.095	–0.126	–0.064	<0.001
**Interaction: Age group and self-reported health**								
16–19/Good	–				–			
20–24/Poor	–0.073	–0.175	0.030	0.164	0.028	–0.053	0.110	0.497

25–29/Poor	–0.133	–0.232	–0.033	0.009	–0.075	–0.155	0.005	0.066
30–34/Poor	–0.031	–0.129	0.066	0.529	–0.038	–0.114	0.038	0.333
35–39/Poor	–0.123	–0.217	–0.029	0.010	–0.035	–0.110	0.040	0.361
40–44/Poor	–0.116	–0.209	–0.023	0.014	–0.063	–0.138	0.013	0.104
45–49/Poor	–0.166	–0.255	–0.076	<0.001	–0.075	–0.148	–0.001	0.046
50–54/Poor	–0.095	–0.184	–0.006	0.037	–0.109	–0.182	–0.036	0.003
55–59/Poor	–0.154	–0.242	–0.066	<0.001	–0.106	–0.178	–0.033	0.004
60–64/Poor	–0.196	–0.285	–0.106	<0.001	–0.113	–0.187	–0.039	0.003
65–69/Poor	–0.140	–0.228	–0.052	0.002	–0.094	–0.168	–0.020	0.012
70–74/Poor	–0.106	–0.194	–0.017	0.019	–0.024	–0.098	0.049	0.518
75–79/Poor	–0.132	–0.223	–0.040	0.005	–0.027	–0.103	0.049	0.482
80–84/Poor	–0.117	–0.213	–0.022	0.016	–0.021	–0.102	0.059	0.601
85–89/Poor	–0.134	–0.239	–0.029	0.012	–0.003	–0.093	0.087	0.952
90+/Poor	–0.072	–0.206	0.062	0.292	–0.030	–0.143	0.083	0.603

Adjusted R-squared for males: 0.296
Adjusted R-squared for females: 0.350
